# Splenetic—Let the Galled Jade Wince

**Published:** 1886-06

**Authors:** 


### SPLENETIC.

" Let the galled jade wince."
— SHAKESPEARE.

For inaccuracy and looseness of statement, and for perversion of facts, commend us to the *New York Medical Record*. Indeed, this agitator leader has become quite notorious for misstatement of facts, and for making "mountains out of mole-hills." In support of this assertion we cite our readers to the *Record's* entire record during the controversy over the organization of the International Medical Congress.

If this shortcoming were the result of ignorance of the subject which the *Record* undertakes to expound, it would be almost inexcusable; but when it is apparently intentional, and for the purpose of attempting to be "smart" at the expense of others,—if it is willful and conscious perversion, it cannot be too severely condemned. A recent case in point has occurred which has very much the latter aspect.

In the *Record* of the 15th of May, in an editorial headed "Serious Split in the American Medical Association," the dissatisfaction and withdrawal of half dozen or so individuals who were not permitted to ride rough-shod over law, order and precedent—the illegally returned delegates, who were very properly not permitted to sit in convention, is magnified into a "serious split in the ranks;" and the object is apparently in keeping with all former exagerations and misrepresentations—to damage the American Medical Association and the cause of the Congress. (We had occasion to correct a misstatement of fact on the part of "our esteemed" only last month, with reference to laparotomy for intestinal wounds.

Really, we had believed the *Record* would show some magnanimity, and take defeat of its revolutionary schemes and agitation with a better grace; and that it would cease to show its teeth, and to vent its impotent spleen on all occasions, because defeated in this Congress business, of which the *Record* seems to have "made a specialty." On the contrary, the *Record* seems to be the Conkling of the Medical Press, and to have been, like the genuine Conkling, "born sneering."

In its issue of May 15th, the *Record*, in an editorial on "The

Pictorial Spirit of the American Medical Association," speaking of, and ridiculing the "beautiful pictures" as the quaint Quimby called those of delegates which adorned the pages of the St. Louis papers during the Convention; and after enumerating the States whose delegates were so complimented—pointing out with especial emphasis the fact that New York was slighted—(notwithstanding Dr. Hammond's picture—the only New Yorker so complimented—was conspicuous amongst the "distinguished;"—may be there is where the shoe pinches—Hammond and not Shrady) says of Texas: "The great State of Texas, which produces annually, millions of bales of cotton, or some other equally valuable products, had only one pictorial representative, an injustice which we trust will be properly commented upon by our fiery contemporary at Austin."

"Our fiery contemporary at Austin" has only to say, by way of comment, that as there were portraits of four Texas physicians, including that same fiery contemporary, in the *Globe-Democrat* on the 5th of May, and one of another Texas physician in the *Republican*, later, the f. c. at A. has nothing to complain of on the score of injustice.

The *Record* man, therefore, is in ignorance of the subject he attempts to elucidate—or—or has made a misstatement of facts as usual, in order to get off a silly sneer at us, and at Texas. "Our esteemed contemporary" evidently feels slighted that HIS phiz was not amongst the distinguished representatives of American Medicine, "an injustice" made the more manifest by the paraded claims that the medical profession of America reside solely in New York and Philadelphia. Really the *Record* should not show such ill nature as to envy the brethren so poor a compliment. We feel sorry for the *Record*—sorry that it should be driven to such straights; but it is the result of having espoused a bad cause; and we commend to "our esteemed," by way of friendly warning and advice, the fate of Annanias,—and the fame of Jo Mulhatton. Surely the *Record* would not emulate either of those worthies.

We should explain that the almost imperceptible sneer at the production of cotton in Texas, is for our benefit; directed at us, in reference to a speech we had the temerity to make at New Orleans in opposition to the nefarious and arrogant scheme to ignore the South in the organization of the International Congress. In urging the claims of the Profession of the South to representation, we spoke of the greatness and wealth of Texas incidentally; and directly of the intelligence and ability of her medical men, and demanded representation in their behalf; and that shot which went home, and which aided largely no doubt in the "destruction of the shenanegan" which the immaculate tried to play on the South and West, seems to be rankling in the bosom of the *Record* still,—which is why we remark "let the galled jade wince."

